# Visualization of SpoVAEa Protein Dynamics in Dormant Spores of *Bacillus cereus* and Dynamic Changes in Their Germinosomes and SpoVAEa during Germination

**DOI:** 10.1128/spectrum.00666-22

**Published:** 2022-05-11

**Authors:** Yan Wang, Norbert O. E. Vischer, Demi Wekking, Alessandra Boggian, Peter Setlow, Stanley Brul

**Affiliations:** a Molecular Biology and Microbial Food Safety, Swammerdam Institute for Life Sciences, University of Amsterdam, Amsterdam, The Netherlands; b Department of Molecular Biology and Biophysics, UConn Health, Farmington, Connecticut, USA; Pennsylvania State University

**Keywords:** *Bacillus cereus*, spores, germinant receptor, SpoVAEa, GerR, GerD, germination

## Abstract

Bacillus cereus spores, like most *Bacillus* spores, can survive for years and germinate when their surroundings become suitable, and germination proteins play an important role in the initiation of germination. Because germinated spores lose the extreme resistance of dormant spores, information on the function of germination proteins could be useful in developing new strategies to control B. cereus spores. Prior work has shown that (i) the channel protein SpoVAEa exhibits high-frequency movement in the outer leaflet of the inner membrane (IM) in dormant B. subtilis spores and (ii) the formation of the foci termed germinosomes between two germination proteins, the germinant receptor GerR and the scaffold protein GerD, in developing B. cereus spores is slower than foci formation by GerR and GerD individually. However, the movement dynamics of SpoVAEa in B. cereus spores, and the behavior of the germinosome upon B. cereus spore germination, are not known. In this study, we found that SpoVAEa fluorescent foci in dormant B. cereus spores move on the IM, but slower than in B. subtilis spores, and they likely co-localize transiently with GerD-mScarlet-I in the germinosome. Our results further indicate that (i) the expression of GerR-SGFP2 and SpoVAEa-SGFP2 with GerD-mScarlet-I from a plasmid leads to more heterogeneity and lower efficiency of spore germination in B. cereus, and (ii) germinosome foci observed by Fluorescence resonance energy transfer (FRET) between GerR-SGFP2 and GerD-mScarlet-I can be lost soon after the spore-phase transition. However, this is not always the case, as some GerR-SGFP2 and GerD-mScarlet-I foci continued to exist, co-localize, and even show a weak FRET signal. These data highlight the heterogeneous behavior of spore germination protein complexes and indicate that some complexes may persist beyond the initiation of germination.

**IMPORTANCE** Bacillus cereus is commonly present in soil and infects humans via contaminated food. In this study, we used B. cereus spores to investigate the movement of the spore-specific inner membrane (IM) channel protein SpoVAEa, the interaction between SpoVAEa and the germinosome scaffold protein GerD, and the dynamics of germinosomes with GerR and GerD in spore germination. Our results expand upon observations of interactions between specific B. cereus spore germination proteins, in particular the GerR germinant receptor A, B, and C subunits and GerD, as well as those between SpoVAEa and GerD. The approaches used in this work could also be used to examine the interactions between GerD and SpoVAEa and other germination proteins in spores of other *Bacillus* species.

## INTRODUCTION

Bacillus cereus is a Gram-positive, rod-shaped, spore-forming bacterium found in soil. The vegetative cells of B. cereus can form endospores under harsh environmental conditions; spores are very resistant and can survive for years due to spore-specific features (1). These spore-specific properties also lead to major challenges to food safety once B. cereus contaminates foods, for example, dairy products, rice, and chilled foods (2, 3). Two major specific structural features of spores include the spore core, containing chromosomal DNA, and the IM, where germinant receptors (GRs) are located, along with the GerD protein and SpoVA protein channels for the major core small molecule, a 1:1 chelate of Ca^2+^ and dipicolinic acid (CaDPA) (4).

Dormant spores can initiate germination when GRs sense specific nutrient germinants in the environment, such as amino acids, inosine, and sugars, and germinated spores then outgrow into vegetative cells. Previous work has indicated that GerD acts as a scaffold protein in localizing GRs in the B. subtilis spore IM in a complex called a germinosome (5, 6), and the GerR GR has been shown to interact with GerD in germinosomes in B. cereus spores (7). Another important group of germination proteins are the SpoVA proteins encoded by the *spoVA* operon, which make up a CaDPA channel in the spore IM. The SpoVA proteins in B. subtilis spores are SpoVAA, SpoVAB, SpoVAC, SpoVAD, SpoVAEb, SpoVAEa, and SpoVAF, encoded in one operon and expressed only in developing spores (8). B. cereus has an operon encoding the same 7 SpoVA proteins, as well as another operon encoding only SpoVAC, SpoVAD, and SpoVAEb. Previous work has indicated that SpoVAEa in B. subtilis spores, a soluble protein on the outer IM surface, moves rapidly around the IM (9–11). It is thus possible that SpoVAEa of B. subtilis spores, perhaps activated in some fashion by stimulated GRs in germinosomes, could trigger the opening of the SpoVA channel, thereby allowing CaDPA release, an early step in spore germination (9–11). Recent work in our lab has shown that in B. subtilis spores, SpoVAEa fused to GFP and expressed from the chromosome is present in only one focus which exhibits random high-frequency movement on the spore IM (11). However, SpoVAEa and the germinosome scaffold GerD protein did not interact in pulldown assays using extracts from B. subtilis spores (Y-Q. Li and B. Hao, unpublished data [9]). Therefore, strong interaction between these proteins has not been observed, although we cannot exclude the existence of transient interactions. There is also no knowledge of the location and physical state of SpoVAEa in B. cereus spores, nor of whether SpoVAEa and GerD proteins co-localize, even transiently.

The spore germination process in bacilli and clostridia has been reviewed in past years (12–14). Initially, germinants bind to GRs, followed by the large-scale release of monovalent cations and CaDPA release via the SpoVA protein channel (15, 16). The kinetics and heterogeneity of spore germination triggered by l-alanine have been analyzed using phase-contrast and fluorescence microscopy, giving the frequency distribution at both the population level and in individual spores of B. cereus strain T (17–19). Previous work using fluorescent reporter protein fusions and the membrane dye FM 4–64 has shown that the B. cereus GR GerR is in the spore IM, and the GerR GR is primarily responsible for l-alanine germination of these spores (7, 20, 21). Recent work has also shown that GerR and GerD could be both be visualized in B. cereus spores using fluorescent reporter proteins, and this work suggested that the formation of germinosome foci was significantly slower than the formation of GerR-SGFP2 and GerD-mScarlet-I foci, with significant heterogeneity in the formation of germinosome foci (7). However, there is little information about the behavior of these germination proteins during B. cereus spore germination.

Strongly enhanced green fluorescent protein (SGFP2) and mScarlet-I have been successfully used to visualize the germination proteins GerR and GerD in spores of B. cereus ATCC 14579 when fluorescent fusion proteins were expressed from a low-copy number plasmid (7, 20). In this work, we aimed to visualize the movement of SpoVAEa fused to SGFP2 in dormant spores of B. cereus ATCC 14579 using fluorescence microscopy, and to analyze the fluorescence distribution by changes in the full width at half-maximum (FWHM) of the fluorescence. Additionally, the phase-contrast intensity and the fluorescence changes of germinosome foci formed by GerR-SGFP2 and GerD-mScarlet-I and by SpoVAEa-SGFP2 and GerD-mScarlet-I were tracked by a time-lapse microscope equipped with phase-contrast and fluorescence analysis options. This work showed that SpoVAEa-SGFP2 foci, one or multiples per spore, exhibited random movements in the IM, and often co-localized with GerD-mScarlet-I in B. cereus spores. The results also suggested that expression of GerR and SpoVAEa proteins with GerD led to slower and more heterogeneous spore germination. Upon the addition of germinant to spores and the initiation of germination, the intensities of germinosome FRET foci were lost most quickly, followed by decreases in the intensities of GerR-SGFP2 and then GerD-mScarlet-I foci. However, some GerR-SGFP2 and GerD-mScarlet-I foci continued to exist and remained co-localized even after the spores transitioned from phase bright to dark, suggesting that germinosome-like complexes may persist beyond the completion of germination. Loss of SpoVAEa-SGFP2 fluorescence intensity also occurred, beginning upon spore transition from phase bright to phase dark.

## RESULTS

### Movement of SpoVAEa foci in dormant spores of B. cereus.

**FIG 1:**
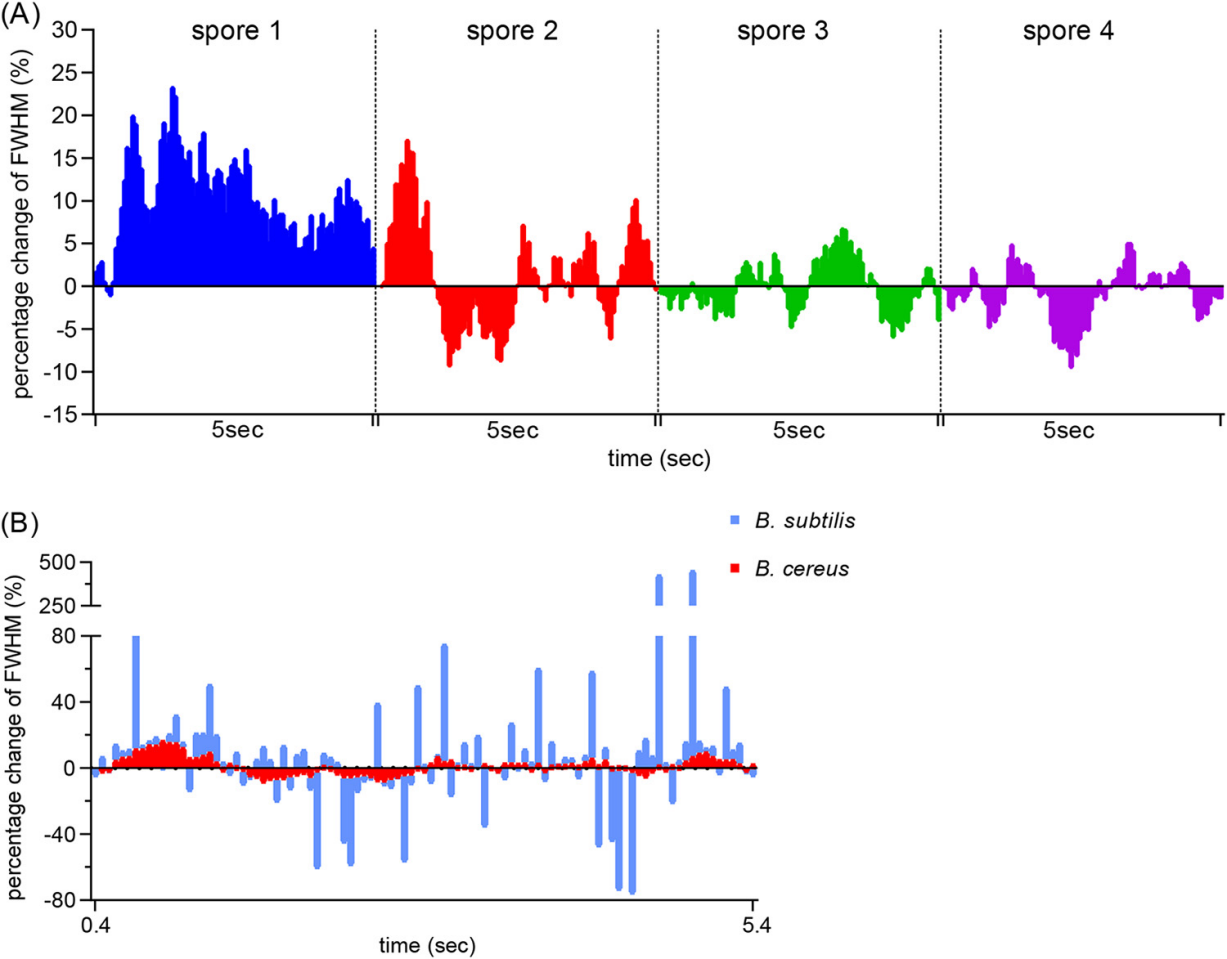
Comparison of the movements of SpoVAEa-SGFP2 foci in dormant spores of B. cereus and B. subtilis. (A) Percentage changes of full width at half-maximum (FWHM) in individual B. cereus spores. (B) Percentage changes of FWHM in B. cereus spore 2 (red squares) and in a B. subtilis spore expressing SpoVAEa-SGFP2 from the chromosome (blue). Raw data of B. subtilis were adapted from a previous work (11). Positive and negative percentages of columns indicate increases and decreases in fluorescence FWHM distribution. A montage of 100 frames (A1 to J10) of B. cereus spore 2 is given in Fig. S1 in the supplemental material.

Previous studies have shown that FWHM can be used to quantitate the fluorescence distribution of spore proteins (11). In this study, we used dormant spores of strain 014 expressing SpoVAEa-SGFP2 from a plasmid to observe the movement of SpoVAEa-SGFP2 foci. The percent changes of FWHM in 100 frames of 4 individual spores over a 5-s period were calculated as up (positive percentage) or down (negative percentage) compared to the first frame. This work showed that SpoVAEa-SGFP2 indeed exhibited what appeared to be random movements or flexing (Fig. 1A, Fig. S1 in the supplemental material). However, the percent changes in the SpoVAEa-SGFP2 FWHM in B. subtilis spores exhibited a wider boundary and higher frequency changes, either up or down, compared to B. cereus spore 2 (Fig. 1B). This result suggested that the SpoVAEa-SGFP2 foci in individual spores of B. cereus and B. subtilis moved in the IM and thus potentially could interact with germinosome components. Of note, SpoVAEa fluorescent foci in B. subtilis spores redistributed at a higher frequency than those in B. cereus spores. This difference may be because different species with a different protein complement are being compared, and with genomic expression of the fluorescent fusion protein in B. subtilis versus expression from a plasmid in B. cereus.

### SpoVAEa-SGFP2 levels are enhanced by GerD expression in recombinant *B. cereus* spores.

**FIG 2:**
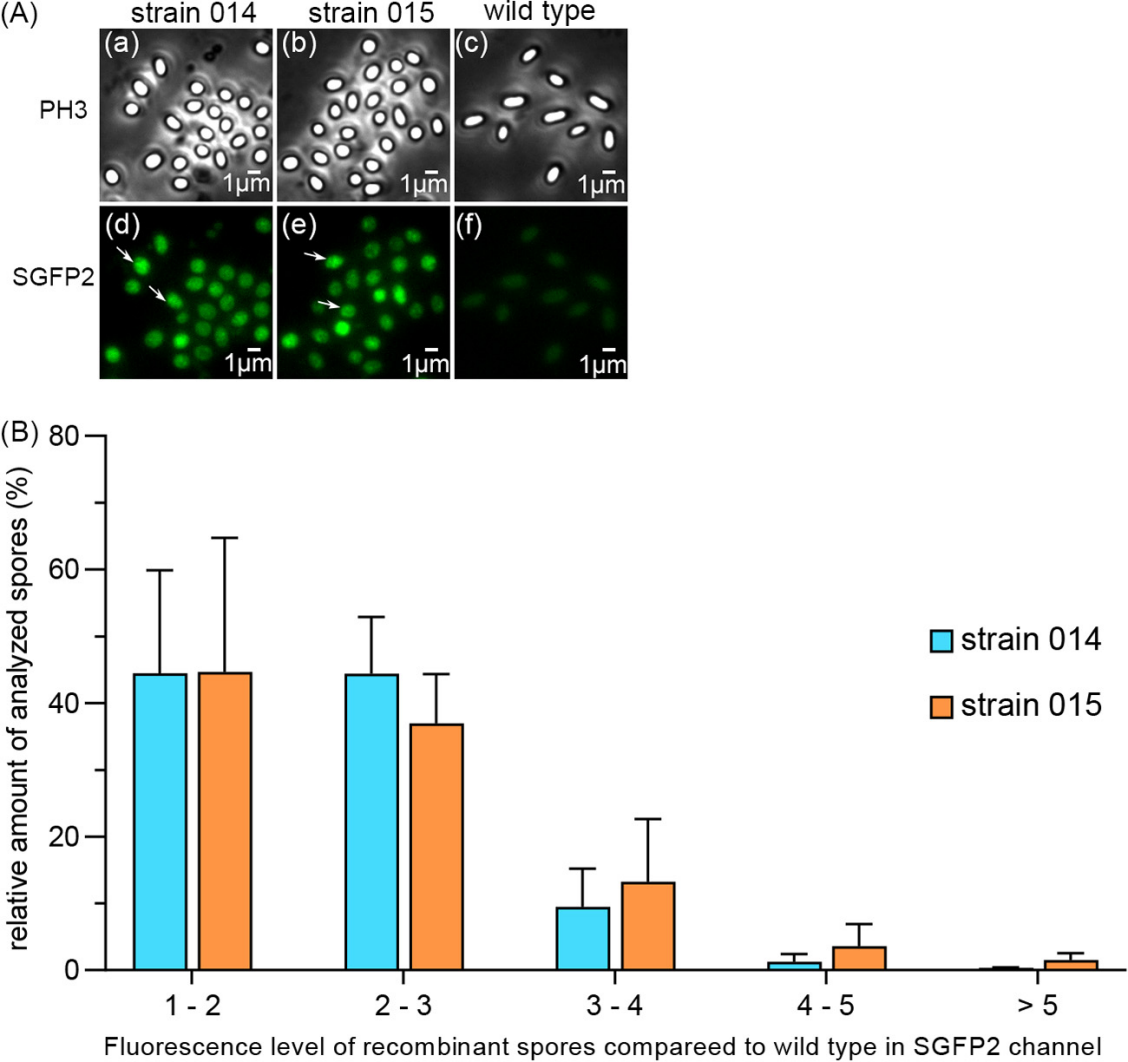
Visualization and comparison of SpoVAEa-SGFP2 fluorescence in dormant spores of B. cereus strains 014 and 015. (A) Dormant spores of strain 014 expressing SpoVAEa-SGFP2, strain 015 expressing SpoVAEa-SGFP2 and GerD-mScarlet-I, and the wild type were visualized: subpanels a, b, and c in the phase-contrast (PH3) channel; panels d, e, and f in the strongly enhanced green fluorescent protein (SGFP2) fluorescence channel. Note the multiple SpoVAE-SGFP2 foci in individual 014 and 015 spores. The white arrows in subpanels a and b indicate representative individual spores. (B) Fluorescence levels in the SGFP2 channel in strain 014 and 015 spores compared to that in wild-type spores. Data are shown as means and standard deviation (SD).

Our recent work showed that GerR and GerD foci are present and co-localized in germinosomes of B. cereus spores (20). In this work, the fusion protein SpoVAEa-SGFP2 was expressed alone in spores of strain 014, or with GerD-mScarlet-I in spores of strain 015. The fluorescence intensities of SGFP2 in strains 014 and 015 were, as expected, both higher than those of wild-type B. cereus spores without the recombinant proteins. However, it was notable that the total fluorescence intensity was significantly higher in spores of strain 015 than in spores of strain 014 (*P* < 0.0001) (Fig. 2, data not shown). In addition, when different fluorescence levels in the spores of recombinant strains 014 and 015 were compared, the distribution of spore population fluorescence intensities, ranging from 3 to over 5-fold the wild-type level, was skewed to the right in strain 015 spores (Fig. 2B). This observation suggests that when SpoVAEa-SGFP2 and GerD-mScarlet-I are expressed from the same plasmid, GerD may contribute to SpoVAEa stability or perhaps enhance SpoVAEa expression.

### Analysis of co-localization of SpoVAEa and GerD proteins.

**FIG 3:**
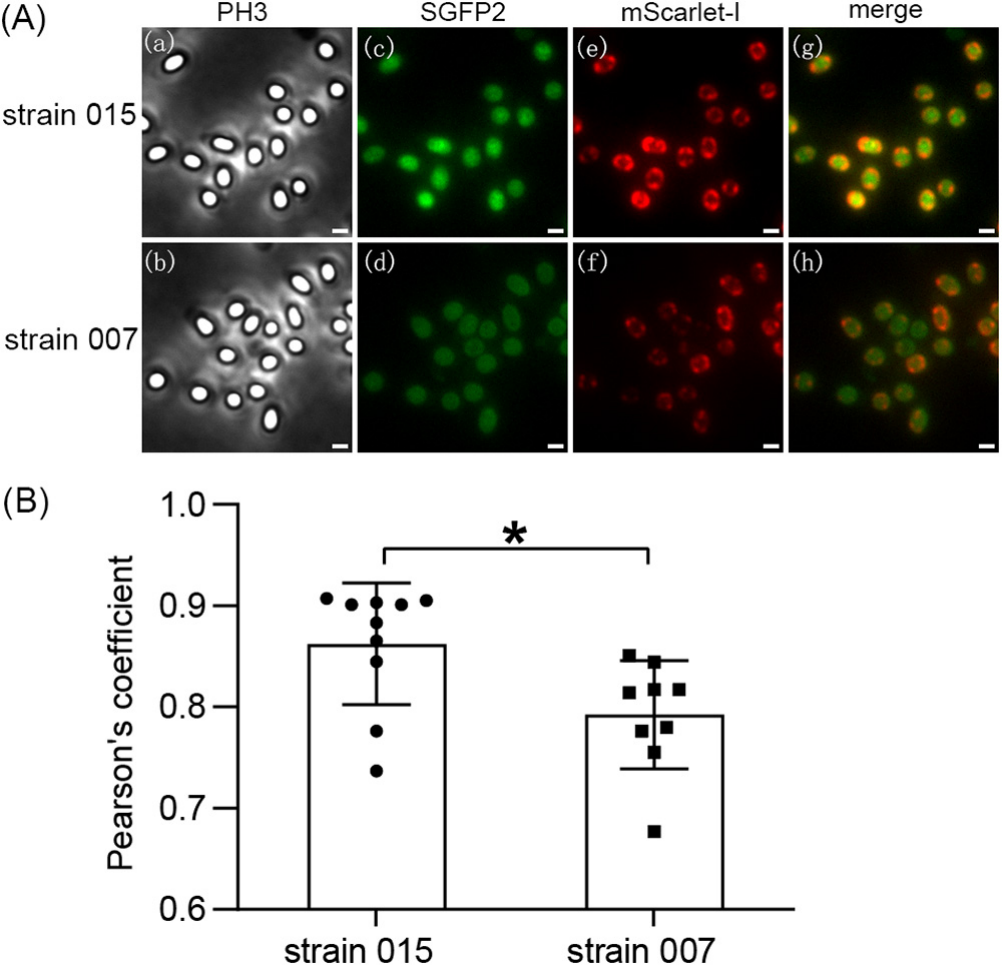
Analysis of co-localization of SpoVAEa and GerD proteins in spores of B. cereus strain 015. (A) Images of strain 015 spores expressing SpoVAEa-SGFP2 and GerD-mScarlet-I and strain 007 spores expressing only GerD-mScarlet-I: subpanels a and b, PH3 channel; subpanels c and d, SGFP2 channel; subpanels e and f, mScarlet-I channel; subpanels g and h, merged image of SGFP2 and mScarlet-I channels. Scale bar is 1 μm. (B) Pearson’s coefficient between SGFP2 and mScarlet-I channels. Data are shown as means and SD. *, *P* < 0.05.

Given the results described above, it was important to examine possible interactions between SpoVAEa-SGFP2 and GerD-mScarlet-I in dormant spores of B. cereus strain 015. The spectra of SGFP2 and mScarlet-I have an overlap that may produce a larger Pearson’s coefficient, a commonly used co-localization indicator (7, 22, 23). To reduce the effects of GerD-mScarlet-I itself, spores of strain 007 expressing GerD-mScarlet-I alone were used as a control. The analysis (Fig. 3) showed that the Pearson’s coefficient of SGFP2 and mScarlet-I channels in spores of strain 015 was significantly higher than those in the control. This result indicated that there is likely co-localization, albeit perhaps only transiently, between SpoVAEa-SGFP2 and GerD-mScarlet-I proteins in spores.

### Expression of GerR and SpoVAEa with GerD affects *B. cereus* spore germination.

**FIG 4:**
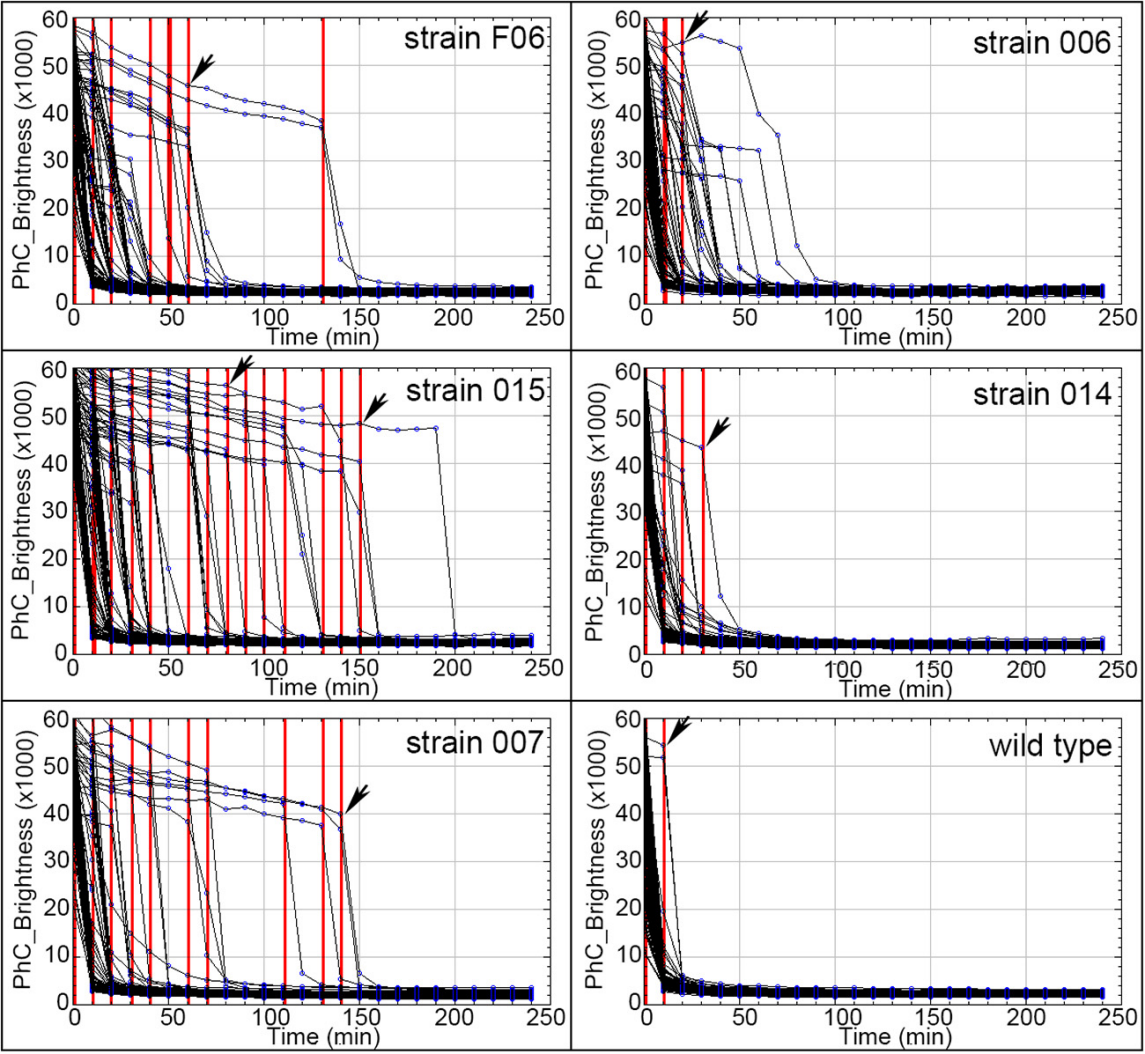
Phase plots show the germination of spores of B. cereus strain F06 expressing GerR-SGFP2 and GerD-mScarlet-I, strain 006 expressing GerR-SGFP2, strain 015 expressing SpoVAEa-SGFP2 and GerD-mScarlet-I, strain 014 expressing SpoVAEa-SGFP2, strain 007 expressing GerD-mScarlet-I, and the wild type. Red lines and black arrows indicate times of initiation of spore germination (germX) for individual spores. For all strains, each black line indicates the change of phase-contrast intensity in an individual spore during germination. The numbers of spores analyzed for strains F06, 006, 015, 014, 007 and the wild type were 108, 92, 122, 233, 107, and 265, respectively.

**FIG 5:**
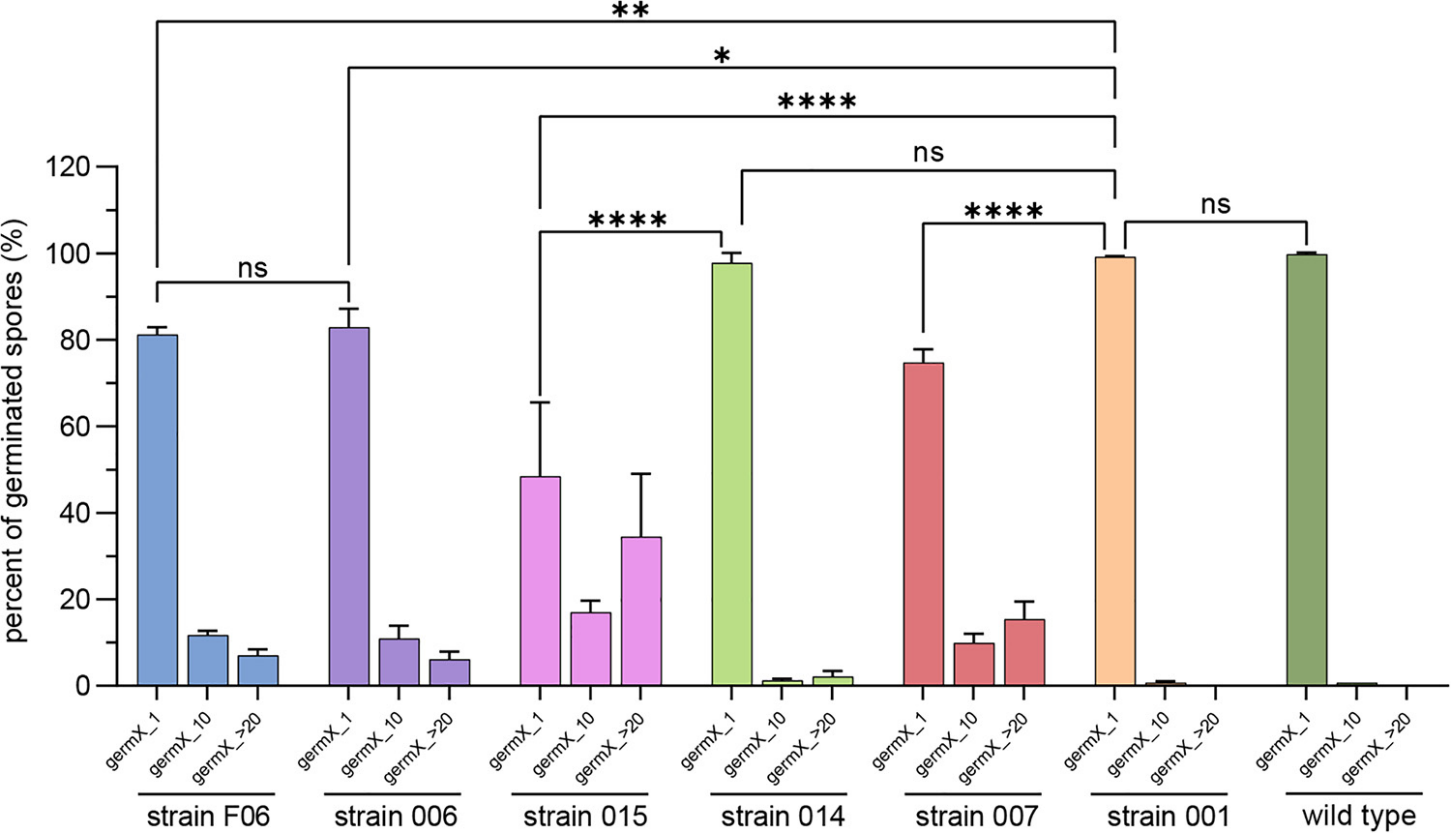
Germination of spores of B. cereus strain F06 expressing fusion proteins GerR-SGFP2 and GerD-mScarlet-I, strain 006 expressing fusion protein GerR-SGFP2, strain 015 expressing fusion proteins SpoVAEa-SGFP2 and GerD-mScarlet-I, strain 014 expressing SpoVAEa-SGFP2, strain 007 expressing fusion protein GerD-mScarlet-I, strain 001 harboring empty plasmid pHT315, and the plasmid-less wild type was examined and different germX times determined. germX_1, initiation of germination at ≤1 min; germX_10, initiation at <10 min; germX_>20, initiation at >20 min. Data are shown as means and SD and are averages of three independent experiments. The numbers of germinated spores for different B. cereus strains analyzed are listed in Table S2 in the supplemental material. ns, not significant; *, *P* < 0.05; **, *P* < 0.01; ****, *P* < 0.0001.

In this work, the time of germination initiation was termed “germX,” defined as the time of the beginning of the rapid decrease in spore phase-contrast image intensity. When GerR-SGFP2 alone or both SpoVAEa-SGFP2 and GerD-mScarlet-I were expressed from a plasmid in strains F06 or 015, respectively, germX values exhibited greater heterogeneity than that in spores of strains 006 and 014 (Fig. 4 and 5). Notably, the germX values for spores of strain 007 expressing GerD-mScarlet-I alone from a plasmid exhibited somewhat more heterogeneity than those of spores of strains 006 and 014 expressing only GerR-SGFP2 or SpoVAEa-SGFP2, respectively (Fig. 4 and 5). These results suggested that expression of GerD-mScarlet-I from a plasmid in B. cereus can lead to increased heterogeneity in spore germination.

Previous work has shown that expression of the l-alanine-responsive GerA GR, controlled by the strong forespore-specific *sspB* promoter in the B. subtilis genome, can significantly increase the rate of germination triggered by l-alanine (24). Our results showed that the spores of all B. cereus strains started to germinate by 1, 10, or >20 min, and these were termed groups germX_1, germX_10, and germX_>20. (Fig. 4 and 5). More than 75% of all spores were in the germX_1 group, except for 015 spores expressing both SpoVAEa-SGFP2 and GerD-mScarlet-I (Fig. 5). Importantly, there was no significant difference in the levels of the germX_1 group between spores of strain 001 containing an empty pHT315 plasmid and plasmid-less wild-type spores. Notably, plasmid expression of fusions of GerR with or without GerD in strains F06 and 006 led to significantly slower germination compared to that of strain 001. This suggests that increased GerR expression may not increase rates of spore germination with l-alanine. However, this is only a suggestion, since (i) the levels of GerR-SGFP2 and of GerR itself are not known in these spores and (ii) it is not known whether GerR-SGFP2 can function in spore germination or might even exert a dominant negative effect on l-alanine germination, although GerR-SGFP2 is certainly competent in germinosome formation. The germination results further showed that plasmid expression of SpoVAEa alone in spores of strain 014 had no significant effect on germination efficiency. However, expression of GerD with SpoVAEa fusion proteins in spores of strain 015 significantly (*P* < 0.0001) slowed germination compared to that in spores containing empty plasmid or spores expressing only one of the fusion proteins (Fig. 4 and 5). Notably, expression of GerR-SGFP2 alone or together with GerD-mScarlet-I in strains 006 or F06 led to a significantly lower germination efficiency compared to that of strain 001 spores (Fig. 5). However, F06 and 006 spore germination was not significantly faster than that of strain 007 spores which expressed the GerD fusion protein alone (data not shown).

Another important piece of information from the results in Fig. 5 is that spores of strain 014 containing only SpoVAEa-SGFP2 exhibited minimal, if any, change in germination from that of wild-type spores. The importance of this result is that recent work (25) has shown that B. cereus spores of strains carrying the plasmid used in this work exhibited a significantly altered protein composition compared to that in plasmid-free spores, with many hundreds of spore proteins significantly up- and downregulated. Since the plasmid backbone in all constructs used in this work is the same, the fact that spores of strains 014 and 001 and plasmid-less wild-type spores exhibited almost identical germination profiles indicates that the presence of the plasmid backbone alone does not alter spore germination. Thus, the effects of plasmids containing fusion proteins on the spore germination kinetics seen in this work can be attributed to the fusion proteins expressed from the plasmids.

### Dynamics of germinosome behavior upon germination triggered by l-alanine in *B. cereus* spores.

**FIG 6:**
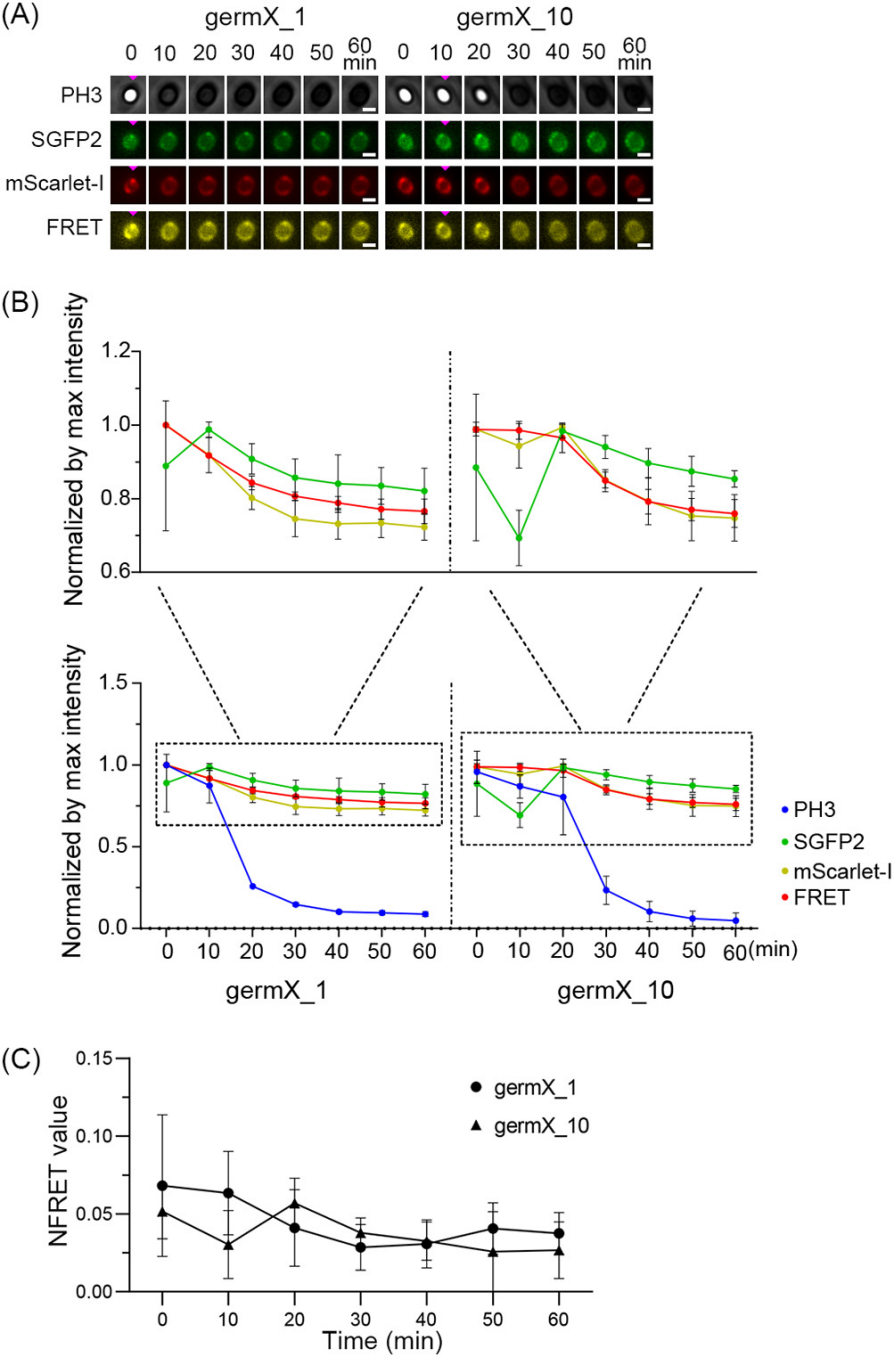
Dynamic changes in germinosome foci upon l-alanine germination of strain F06 spores expressing GerR-SGFP2 and GerD-mScarlet-I. (A) Visualization of changes in GeR-SGFP2, GerD-mScarlet-I, and germinosome foci at 10-min intervals. Left column shows PH3, SGFP2, mScarlet-I, and fluorescence resonance energy transfer (FRET) channels of an individual spore in the germX_1 group. Right column shows PH3, SGFP2, mScarlet-I, and FRET channels of an individual spore in the germX_10 group. Pink triangles indicate times of initiation of germination. Scale bar is 1 μm. (B) Graphs of intensities in the PH3, SGFP2, mScarlet-I, and FRET channels of spore populations. Left column, germX_1 group; right column, germX_10 group. (C) Graph of the dynamics of normalized FRET (NFRET) values in populations of spores of strain F06. Black circles represent the germX_1 group, black triangles represent the germX_10 group. Data are shown as means with SD. The number of germinated spores of B. cereus strain F06 analyzed is listed in Table S2. Statistical analyses of differences between individual time points in graphs compared to the previous one are shown in Table S3 in the supplemental material.

Our recent study suggested that the formation of FRET foci between GerR-SGFP2 and GerD-mScarlet-I could be significantly slower than the formation of foci in the SGFP2 and mScarlet-I channels during B. cereus spore formation (7). In this work, we tracked the dynamic changes in germinosome FRET foci upon germination triggered by l-alanine in spores of strain F06 expressing GerR-SGFP2 and GerD-mScarlet-I (Fig. 6). The phase-contrast channel (PH3) recorded the transition between a phase-bright to a phase-dark spore at 1 or 10 min of germination in the germX_1 and germX_10 groups, respectively (Fig. 6A, Table S3 in the supplemental material). Upon the initiation of phase transition in the germX_1 group, the intensity of germinosome FRET foci fell significantly, but there was no significant decrease in the intensity of GerR-SGFP2 foci (Fig. 6B, Table S3). The average normalized FRET values (NFRET) in strain F06 spores at 10, 20, 30, 40, 50, and 60 min in the germX_1 group were all lower than those at 0 min, but this downward trend was not significant, likely due to the heterogeneity in the germination of individual spores (Fig. 6C). Note that while Fig. 6A shows results for an individual spore, Fig. 6B and C show population averages. When GerR-SGFP2 or GerD-mScarlet-I alone were expressed in spores of strains 006 and 007, the intensities of 006 spores in the SGFP2 channel in the germX_1 group at 20 min were significantly lower than those at 0 min (*P* < 0.01). The intensities of 007 spores in the mScarlet-I channel of the germX_1 group at 20 min were also significantly lower than those at 0 min (*P* < 0.001) (Fig. S2, Table S4). The intensities of the FRET foci of F06 germX_10 spores at 40 min and beyond were also significantly lower compared to those of the 0-min spores. The FRET intensity drop occurred after the initiation of the rapid fall in spore phase contrast image intensity starting at 20 min after the initiation of the experiment (Fig. 6B, Table S3). In addition, the remaining GerD-mScarlet-I foci became less intense in the germinated spores of the germX_1 and germX_10 groups (Fig. 6B). The intensities of strain 006 spores expressing GerR-SGFP2 alone in the SGFP2 channel of the germX_10 group showed a downward trend during our experiment, except for the 30-min time point. The same trend was observed for the 007 spores, although the drop in the intensities of the mScarlet-I channel only began 20 min after the start of the experiment (Fig. S2, Table S4). While these results indicate that the germinosome FRET foci in spores of B. cereus can be lost soon after the spore-phase transition, this is not always the case, as some GerR-SGFP2 foci and GerD-mScarlet-I foci continued to exist, co-localize, and even show a weak FRET signal (compare Fig. 6A left- and right-hand panels). These data highlight the heterogeneous behavior of spore germination protein complexes and indicate that some complexes may persist well beyond the initiation of germination. However, the meaning of this observation, if any, is unclear, and this may be only another example of the heterogeneity seen in spore germination when multiple individual spores are examined.

### Dynamics of SpoVAEa and GerD proteins during germination of *B. cereus* spores triggered by l-alanine.

**FIG 7:**
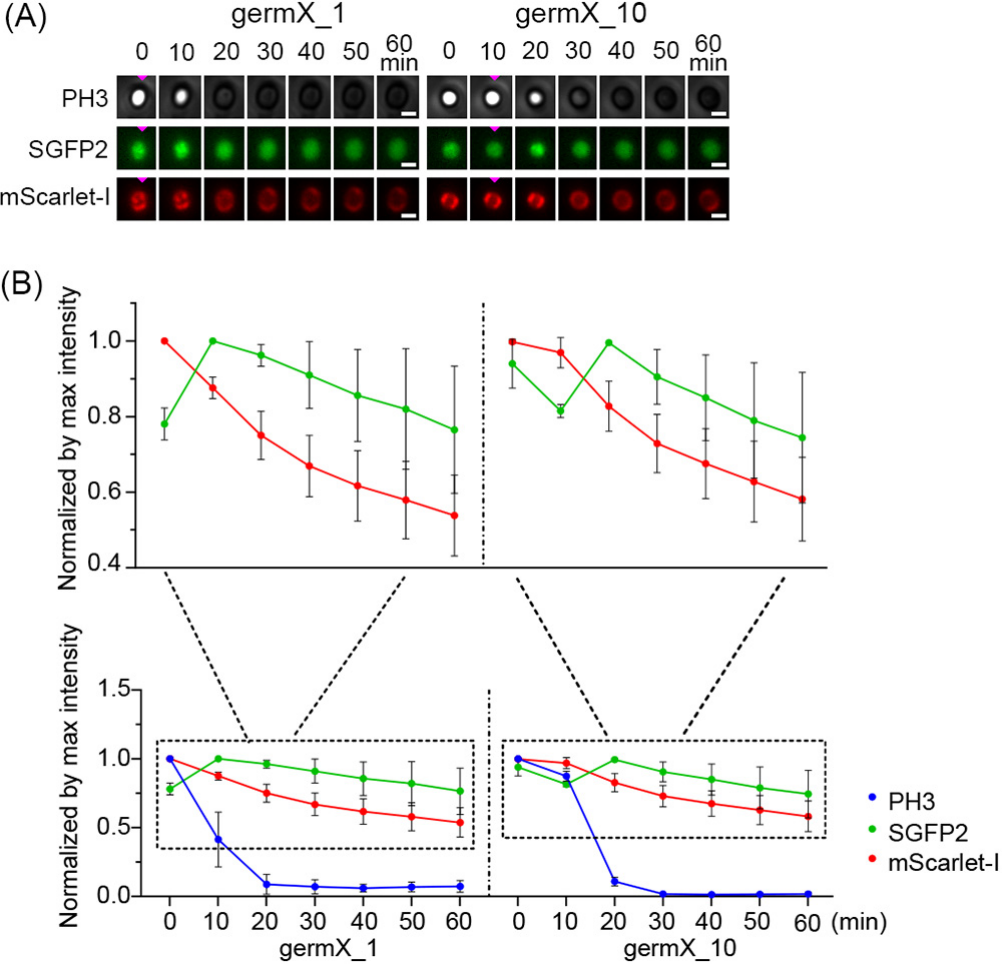
Dynamic changes in SpoVAEa-SGFP2 and GerD-mScarlet-I fluorescence intensities during l-alanine germination of strain 015 spores. (A) Visualization of changes in SpoVAEa-SGFP2 and GerD-mScarlet-I at 10-min intervals in one spore. Left column shows PH3, SGFP2, and mScarlet-I channels of an individual spore in the germX_1 group. Right column shows PH3, SGFP2, and mScarlet-I channels of an individual spore in the germX_10 group. Pink triangles indicate the initiation of germination. Scale bar is 1 μm. (B) Graphs of PH3, SGFP2, and mScarlet-I channels from multiple individual spores. Left column, germX_1 group; right column, germX_10 group. Data are shown as means with SD and represent three independent experiments. The numbers of germinating strain 015 spores analyzed are listed in Table S2. Statistical analysis of values in graphs compared to preceding values is shown in Table S5.

In this work, recombinant spores of strain 015 expressing SpoVAEa-SGFP2 and GerD-mScarlet-I from a plasmid were used to visualize the dynamic changes of SpoVAEa and GerD upon germination initiated by l-alanine. The results showed that the phase-contrast intensity of germinated spores of strain 015 at 10 min in the germX_1 group was greatly decreased compared to that of phase-bright spores at 0 min. In the germX_10 group, the phase transition occurred after 10 min and the phase-contrast intensity of germinated spores at 20 min was decreased compared to that at 0 min (Fig. 7, Table S5). The fluorescence intensity in the SGFP2 or mScarlet-I channels of germinated spores at 10 min in the germX_1 group was decreased compared to that of phase-bright spores at 0 min, but this reduction was not significant (*P* > 0.05). Instead, when SpoVAEa-SGFP2 alone was expressed in strain 014 spores, the fluorescence intensities of strain 014 spores in the SGFP2 channel of the germX_10 group at 10 min were significantly decreased compared to the values at 0 min (Fig. S2, Table S4). These results showed that the SpoVAEa-SGFP2 foci were lost, and overall SGFP2 fluorescence intensity dropped, following initiation of germination. The same is true for GerD-mScarlet-I, although, in accordance with our results described in Fig. 6, some foci continued to exist beyond the phase transition, albeit with lower fluorescent intensity.

## DISCUSSION

B. cereus spores, like most *Bacillus* spores, have various resistance characteristics due to spore-specific structures, and can restart their metabolism only after spore germination is completed. The nutrient germination of spores is initiated by germinant binding to specific GRs localized in the IM in B. cereus spores, with GerR triggering germination with l-alanine (21, 26). SpoVAEa is a component of the IM SpoVA protein channel for CaDPA, and GerD is a scaffold protein playing an important role in germinosome formation and thus in spore germination in B. subtilis and B. cereus (6, 7, 9). To extend the latter observations, we studied the dynamic changes of the SpoVAEa protein in dormant and germinated spores of B. cereus, and the kinetic changes in germinosome foci during the germination process.

Based on previous observations, the expression levels of GerD and most SpoVA proteins in B. subtilis spores are ~10^2^- and 10^3^-fold higher than those of GRs, respectively (27). Fluorescence microscopy of B. cereus spores showed clear foci of GerD-mScarlet-I and less-distinct foci of SpoVAEa-SGFP2; one possible reason for this could be the different expression levels of these two proteins. However, the weaker fluorescent signal of SpoVAEa-SGFP2 compared to that of GerD-mScarlet-I in our work might also be caused by expression of only one subunit of the SpoVA complex alone, or perhaps SpoVAEa has a lower expression level than other SpoVA proteins, as was shown for SpoVAEa levels compared to SpoVAD levels in B. subtilis spores (9).

In this study, recombinant B. cereus spores expressing fluorescent fusions to GerR and SpoVAEa with or without a GerD fusion protein from plasmids were used to assess the effects of these fusion proteins on germination triggered by l-alanine. Plasmid expression of GerD-mScarlet-I alone or with SpoVAEa-SGFP2 clearly slowed spore germination, as shown by increased spore levels in the germX_10 and germX_>20 groups in the spores expressing GerD-mScarlet-I (Fig. 5; Table S2). These results indicated that plasmid expression of the GerD fusion protein has an inhibitory effect on spore germination efficiency and thus increased germination heterogeneity, consistent with the findings of previous work (17, 19). However, spores with plasmid expression of the GerR fusion alone also germinated more slowly than spores containing empty pHT315 plasmid, but coexpression of the GerD fusion did not slow germination any further (Fig. 5, Table S2). The reasons for the effects of the various fusion proteins on rates of spore germination are not clear. Two possible reasons are that the protein fusions are either not functional in germination or that they exert a dominant negative effect on the function of the wild-type proteins. While the latter is a possibility, the GerD and GerR fusion proteins do form germinosomes (7), so at least some wild-type function is retained. In addition, GerD and GR fusion proteins analogous to those used in the current work were functional in B. subtilis spore germination (28). However, ultimately answering this functionality question definitively will require analysis of the effects of the fusion proteins in the appropriate null nutant backgrounds. Notably, a recent study suggested that in B. subtilis, the B subunit of the GerA GR, GerAB, is responsible for binding the germinant l-alanine, and this is consistent with molecular dynamics analyses of l-alanine binding to GerAB (29). Our recent studies have also suggested that there is a very close interaction between GerRB and GerD in the germinosome (7). Consequently, the reason for the inhibitory effect of plasmid GerD-mScarlet expression plus or minus SpoVAEa-SGFP2 on spore germination could be that excess GerD can occupy or occlude the l-alanine binding sites on GerRB, while plasmid expression of GerR-SGFP2 would increase GerRB subunit levels such that the concomitant expression of the GerD protein fusion has minimal effects on spore germination.

**FIG 8:**
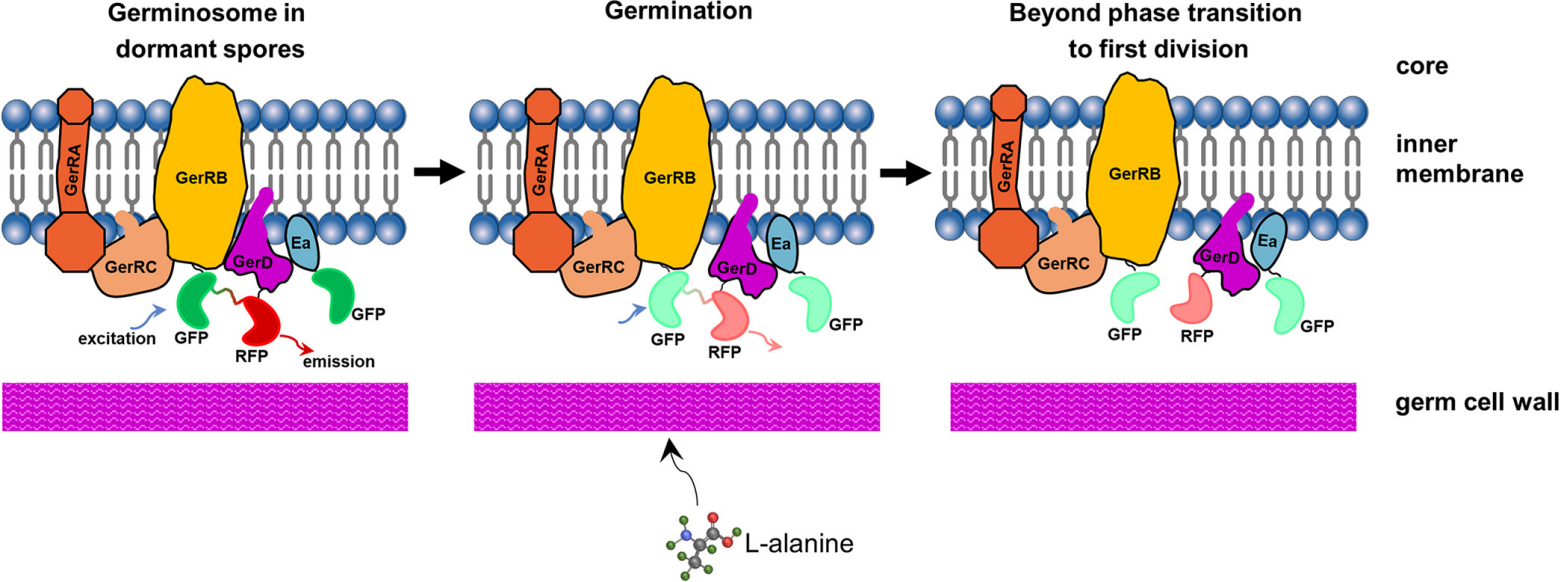
A proposed model of germinosome dynamics during germination triggered by l-alanine in B. cereus spores. Left panel shows (i) FRET-positive germinosome formation due to close interaction between GerR-SGFP2 and GerD-mScarlet-I, with the darker green-red line between GFP (SGFP2, deep green) and RFP (mScarlet-I, deep red) indicating the energy transfer path in the FRET event between GerR and GerD; and (ii) likely, albeit transient, co-localization between SpoVAEa and GerD proteins. Ea, SpoVAEa. Middle panel shows that (i) FRET-positive germinosomes may be lowered in intensity following the phase transition in germination initiation caused by l-alanine: the FRET signal (light green-red line) between GFP (light green) and RFP (light red) has become weak, indicating that close interaction between GerR and GerD has been gradually lost, consistent with GerD-mScarlet-I and GerRB-SGFP2 moving apart; and (ii) SpoVAEa-SGFP2 and GerD-mScarlet-I fluorescence intensities have decreased upon initiation of germination. Right panel shows (i) hypothesized loss of FRET-positive germinosomes; some GerR-SGFP2 foci (light green) and GerD-mScarlet-I foci (light red) may continue to exist, indicated by the co-localization of GerR and GerD after the phase transition; and (ii) some GerD foci also continue to exist and likely co-localize, perhaps transiently with SpoVAEa.

Our recent results, including the use of FRET analysis, on the dynamics of germinosome formation in B. cereus spores suggest that the formation of foci in the FRET channel may be significantly slower than the formation of GerR-SGFP2 and GerD-mScarlet-I foci (7). To further assess germinosome dynamics, we observed the changes in germinosome foci upon germination initiated by l-alanine in B. cereus spores. In this experiment, the protein FRET pairs, GerR-SGFP2 and GerD-mScarlet-I, were expressed from a plasmid and driven by their native promoters during sporulation. Possibly consistent with the role of the B subunit of GerA in B. subtilis, GerRB may also be responsible for initiating germination with l-alanine in B. cereus (Fig. 6). Once the process of spore germination was initiated, our results showed that some GerD-GerR co-localization likely remains, even though FRET-positive germinosome foci were lost after the initiation of germination. Figure 8 shows a hypothetical sequence of events that may occur during spore germination. A note of caution is warranted because the germination proteins analyzed in this work were expressed from a plasmid, and this may disrupt the dynamic balance in germination protein assembly in sporulation and germination. However, all germination proteins studied were expressed from the plasmid under the control of their respective native promoters, allowing relative expression differences to be conserved, although this will likely be influenced by plasmid copy number, which is variable.

In summary, the SpoVAEa-SGFP2 protein exhibits random movement on the outer surface of the spore IM and a likely, at least transient, co-localization with GerD-mScarlet-I in dormant spores of B. cereus; this transient co-localization may be a means of transduction of a signal from a germinosome to the SpoVA channels, triggering CaDPA release from spores. The latter idea is certainly worth studying further. Studying spore germination by phase-contrast microscopy suggested that expression of GerR-SGFP2 or SpoVAEa-SGFP2 with GerD-mScarlet-I from a plasmid leads to more heterogeneity and less efficient spore germination in B. cereus, pointing to the need for future studies to investigate the stoichiometry of the germinosome components in B. cereus in more detail. The dynamics of germination showed that germinosome foci composed of GerR-SGFP2 and GerD-mScarlet-I were lost soon after the phase transition. Further work related to the machinery of spore germination should likely focus on detailed studies of interactions between elements of the SpoVA channel, GerD and GR subunits.

## MATERIALS AND METHODS

### Recombinant plasmids and *B. cereus* strains.

The recombinant plasmids and B. cereus strains used in this study are listed in Table 1. All primers used are listed in Table S1 in the supplemental material. The recombinant plasmids were constructed as described in previous studies (7, 20). Briefly, the 226-bp region located upstream of the *spoVA* operon was considered the promoter region of *spoVAEa* gene and named PEa. The PEa fragment was inserted into pHT315 between *Kpn* I and *Xba* I sites, resulting in plasmid pHT315-PEa. Next, the *spoVAEa* (BC_4065) gene was amplified from genomic DNA of B. cereus ATCC 14579 (GenBank ID: AE016877) using a pair of primers, 315_YW-42 and 315_YW-43. The *SGFP2* gene with stop codons was fused to the 3′ end of the *spoVAEa* gene using a two-fusion PCR. The fusion product was inserted into pHT315-PEa between the *Xba* I and HindIII sites. The resulted ligation product was transformed into competent E. coli cells, and selection of positive clones produced plasmid pHT315-f14. The fusion fragment PD-*gerD*-*mScarlet-I* was amplified from plasmid pHT315-f05 and inserted into pHT315-f14 between the *Kpn* I and EcoR I sites, giving plasmid pHT315-f15. The correct construction of recombinant plasmids pHT315-f14 and pHT315-f15 was confirmed by sequencing, followed by electroporation into competent B. cereus ATCC 14579 cells, and finally by selection and confirmation with colony PCR of an erythromycin-positive single colony.

**TABLE 1:** B. cereus strains and plasmids used in this study^*a*^

B. cereus strain	Plasmid present (+)	Description of inserted genes	Source or reference
ATCC 14579	Wild-type	None	Lab stock
001	+pHT315 Ery^r^	Empty plasmid	20
014	+pHT315-f14 Ery^r^	PEa-*SpoVAEa*-*SGFP2*	This study
015	+pHT315-f15 Ery^r^	PEa-*SpoVAEa*-*SGFP2* and PD-*gerD-mScarlet-I*	This study
006	+pHT315-f01 Ery^r^	PR-*gerR(A-C-B)*-*SGFP2*^*b*^	7
007	+pHT315-f05 Ery^r^	PD-*gerD*-*mScarlet-I*	7
010	+pHT315-f10 Ery^r^	PD-*gerD*-*SGFP2*	7
F06	+pHT315-f06 Ery^r^	PR-*gerR(A-C-B)-SGFP2*^1^ and PD-*gerD-mScarlet-I*	7

a PEa, promoter of *spoVA* operon; PR, promoter of *gerR* operon; PD, promoter of *gerD*; Ery^r^, resistant to erythromycin.

b Note that the SGFP2 is fused to the last gene in the *gerR* operon, *gerRB*, as the order of cistrons in this operon is PR-*gerRA-gerRC-gerRB* (7).

### High-frequency time-lapse image acquisition and analysis.

Spores of B. cereus strain 014 were prepared and purified as described in previous work (20). A Nikon Eclipse Ti-E microscope (Nikon Instruments, Tokyo, Japan) equipped with a sCmos camera (Hamamatsu Flash 4.0 V2, Hamamatsu City, Japan) and wide-field fluorescence components was used to capture 100 frames of 14-bit SGFP2 images (excitation at 488 nm and emission at 535 nm), with a 50-ms exposure time for each frame and no delay interval. Raw data from B. subtilis expressing SpoVAEa-SGFP2 were taken from the wide-field microscopy data described by Wen et al. (11). Individual spores located in 100 frames were selected, duplicated, and analyzed by the plugin Adrian’s FWHM in ImageJ. The percent changes of FWHM in the second frame to the hundredth frame relative to the FWHM in the first frame were calculated and plotted using GraphPad Prism version 9.3 software.

### Images of SpoVAEa-SGFP2 expressed in spores of *B. cereus* strains 014 and 015; acquisition and analysis.

The preparation of B. cereus spores and their visualization were carried out as described in previous work (20). Spores of strain 014 expressing SpoVAEa-SGFP2 and spores of strain 015 expressing SpoVAEa-SGFP2 and GerD-mScarlet-I were captured in the phase-contrast and SGFP2 (excitation at 470 nm and emission at 516 nm) channels using a Nikon Eclipse Ti-E microscope. Images were analyzed by the ObjectJ SporeAnalyzer_1c.ojj in Fiji/ImageJ (https://sils.fnwi.uva.nl/bcb/objectj/examples/SporeAnalyzer/MD/SporeAnalyzer.html). Three independent experiments were performed, and the data were analyzed by GraphPad Prism version 9.3 software.

### Co-localization assays and data analysis.

Spores of B. cereus strains 015 and 007 were prepared and purified as described in previous work (20). Spores of strains 015 and 007 were captured in three channels: phase-contrast, SGFP2, (excitation at 470 nm and emission at 516 nm) and mScarlet-I (excitation at 555 nm and emission at 593 nm), using a Nikon Eclipse Ti-E microscope. All acquired images in the co-localization assay were processed with ImageJ. The SGFP2 and mScarlet-I images were used to calculate the co-localization indicator Pearson’s coefficient with the JACoP plugin in ImageJ (22).

### Germination assays by time-lapse imaging and data processing.

Dormant spores of strains F06, 006, 015, 014, 007, 001 and wild-type spores were prepared and purified as described previously (20). For microscope slide preparation, a 65-μL gene frame with 0.25-mm thickness (Thermo Fisher Scientific, The Netherlands, cat. no. AB0577) was attached on the center of a normal microscope slide. A liquid mixture for an agarose pad was made using a 1:1 mixture of 2× germination buffer (see below) and 2% agarose in a heat block at 55°C. A 60-μL volume of the liquid mixture was pipetted on the area of frame, immediately pressed with another slide, and placed at 4°C for at least 20 min to solidify.

Dormant spores suspended in ice-cold phosphate-buffered saline (PBS) (pH 7.4) were heat-activated for 15 min at 70°C and washed three times with ice-cold PBS (pH 7.4) by centrifugation at 14,300 × *g* for 15 min at 4°C. The heat-treated spores were suspended in ice-cold germination buffer (50 mM Tris-HCl [pH 7.4], 10 mM NaCl, and 100 mM l-alanine) at an OD_600_ (optical density at 600 nm) of 15. The spore suspension (1.3 μL) was dropped onto the solid agarose pad and immediately covered by a cover slip (18 × 18 mm) and was now ready for time-lapse microscopy.

A Nikon Eclipse Ti-E microscope (Nikon Instruments, Tokyo, Japan) equipped with an sCmos camera (Hamamatsu Flash 4.0 V2, Hamamatsu, Japan), phase-contrast, and wide-field fluorescence components was used to track the germination of B. cereus spores for 4 h with 10-min intervals. Spores of strain F06 expressing GerR-SGFP2 and GerD-mScarlet-I were captured by four images: phase contrast, SGFP2 fluorescence (excitation at 470 nm and emission at 516 nm), mScarlet-I (excitation at 555 nm and emission at 593 nm), and FRET (excitation at 470 nm and emission at 593 nm). Spores of strain 006 expressing GerR-SGFP2 and spores of strain 014 expressing SpoVAEa-SGFP2 were captured by phase-contrast and SGFP2 images. Spores of strain 015 expressing SpoVAEa-SGFP2 and GerD-mScarlet-I were captured by phase-contrast, SGFP2, and mScarlet-I images, and spores of strain 007 expressing GerD-mScarlet-I were captured by phase-contrast and mScarlet-I images. Wild-type spores were captured by phase-contrast, SGFP2, mScarlet-I, and FRET images. Spores of strain 001 were captured by phase-contrast images.

All 16-bit type images captured in the germination assays were converted to 32-bit type. Selection and measurement of the background area in samples without images were carried out, and the background was subtracted using the Process–Math–Subtract tool in Fiji/ImageJ. The germinated spores were analyzed, and various intensities of individual spores were measured using the ObjectJ SporeTrackerC_1h.ojj in Fiji/ImageJ (https://sils.fnwi.uva.nl/bcb/objectj/examples/sporetrackerc/MD/SporeTrackerC.html). Fluorescence intensities of plasmid-containing spores which were lower than that of the wild-type spores were considered aberrant values and were not counted. Three independent experiments were performed. The intensities of wild-type spores were subtracted from the intensities of plasmid-containing spores, and the differences were used to perform statistical comparisons with Graph Pad Prism version 9.3 software. The calculation of NFRET (normalized FRET) was performed as described previously (7).
