# Supplementary material for: Visualization of SpoVAEa Protein Dynamics in Dormant Spores of Bacillus cereus and Dynamic Changes in Their Germinosomes and SpoVAEa during Germination
Source: Microbiol Spectr. 2022 May 11;10(3):e00666-22. doi: 10.1128/spectrum.00666-22 (PMC9241617; doi:10.1128/spectrum.00666-22)
Supplement: SUPPLEMENTAL FILE 1 — Supplemental material. Download spectrum.00666-22-s001.pdf, PDF file, 0.3 MB [file spectrum.00666-22-s001.pdf]

Supplementary Materials

TABLE S1. Primers used in this study

Primers	Sequence (5'-3')	Purposes or functions
315_YW-42	GGGGTACCAGGTGTTTCATAATGAGTGG	*Kpn* I-*spoVA* promoter-Fw
315_YW-43	GCTCTAGACTTTCATTCACCCCTTCAC	*Eco*R I-*spoVA* promoter-Rv
315_YW-44	GCTCTAGATTGGTCACAGATGGAGAT	*Xba* I-*spoVAEa*-Fw
315_YW-45	TCCTCGCCCTTGCTCACCATgctgccgctgccgctgcc GGCTTCGTCATAAAAACCA	*spoVAEa-(GS)*$_3$*-SGFP2*-Rv
315_YW-46	TGGTTTTTATGACGAAGCCggcagcggcagcggcagc ATGGTGAGCAAGGGCGAGGA	*spoVAEa-(GS)*$_3$*-SGFP2*-Fw
315_YW13	CCCAAGCTTTTACTTGTACAGCTCGTCCAT	*SGFP2-Hin*d III-Rv
315_YW-47	CAAATGGGGAAGTCTCGT	*spoVAEa*-sequencing-Rv

a. Restriction cleavage sites are underlined.

b. The six-residue flexible linker (Gly-Ser-Gly-Ser-Gly-Ser) coding sequence is written in lower-case letters.

TABLE S2. The numbers of germinated spores of different *B. cereus* strains examined in three independent experiments

B. cereus strains	replication	1min	10min	>20min	total
strain F06	replication 1	74	10	5	89
	replication 2	87	12	9	108
	replication 3	56	9	5	70
strain 006	replication 1	39	7	4	50
	replication 2	43	4	3	50
	replication 3	78	10	4	92
strain 015	replication 1	13	9	23	45
	replication 2	69	18	25	122
	replication 3	48	13	19	80
strain 014	replication 1	35	no	no	35
	replication 2	228	2	3	233
	replication 3	62	1	2	65
strain 007	replication 1	29	3	6	38
	replication 2	82	13	12	107
	replication 3	59	8	16	83
wild type	replication 1	29	no	no	29
	replication 2	23	no	no	23
	replication 3	263	2	no	265

TABLE S3. Statistical comparisons between each timepoint and 0 min of intensities in Fig. 6 of channels from germinating spores of *B. cereus* strain F06

Groups	Comparisons between times[1]	Channels			
		PH3	SGFP2	mScarlet-I	FRET
germX_1	10 min	ns	ns	ns	ns
	20 min	****	ns	ns	*
	30 min	****	ns	ns	***
	40 min	****	ns	***	***
	50 min	****	ns	***	***
	60 min	****	ns	***	****
germX_10	10 min	ns	ns	ns	ns
	20 min	ns	ns	ns	ns
	30 min	****	ns	ns	ns
	40 min	****	ns	*	*
	50 min	****	ns	**	**
	60 min	****	ns	**	**

ns, not significant; *, $P < 0.05$; **, $P < 0.01$, ***, $P < 0.001$; ****, $P < 0.0001$

[1]Comparisons were between intensities at indicated times and the time above, except for the 10 min samples that were compared to spores at time 0.

TABLE S4. Statistical comparison of vertical pairs' intensities in Fig. S2 of channels from germinating spores of *B. cereus* strains 006, 007 and 014.

Groups	Comparisons between times[1]	strain 006	strain 007	strain 014
		SGFP2 channel	mScarlet-I channel	SGFP2 channel
gerX_1	10 min	ns	ns	**
	20 min	**	***	***
	30 min	***	****	**
	40 min	****	****	*
	50 min	****	****	ns
	60 min	****	****	ns
germX_10	10 min	ns	ns	ns
	20 min	*	ns	ns
	30 min	ns	**	ns
	40 min	ns	****	ns
	50 min	ns	****	ns
	60 min	ns	****	ns

ns, not significant; *, $P < 0.05$; ***, $P < 0.001$; ****, $P < 0.0001$.

[1]Comparisons were between intensities at indicated times and the time above, except for the 10 min samples that were compared to spores at time 0.

TABLE S5. Statistical comparisons of vertical pairs of intensities in Fig. 7 of channels from germinating spores of *B. cereus* strain 015

Groups	Comparisons between times[1]	Channels		
		PH3	SGFP2	mScarlet-I
germX_1	10 min	****	ns	ns
	20 min	****	ns	ns
	30 min	****	ns	***
	40 min	****	ns	****
	50 min	****	ns	****
	60 min	****	ns	****
germX_10	10 min	ns	ns	ns
	20 min	****	ns	ns
	30 min	****	ns	*
	40 min	****	ns	**
	50 min	****	ns	****
	60 min	****	ns	****

ns, not significant; ****, $P < 0.0001$.

[1]Comparisons were between intensities at indicated times and the time above, except for the 10 min samples that were compared to spores at time 0.

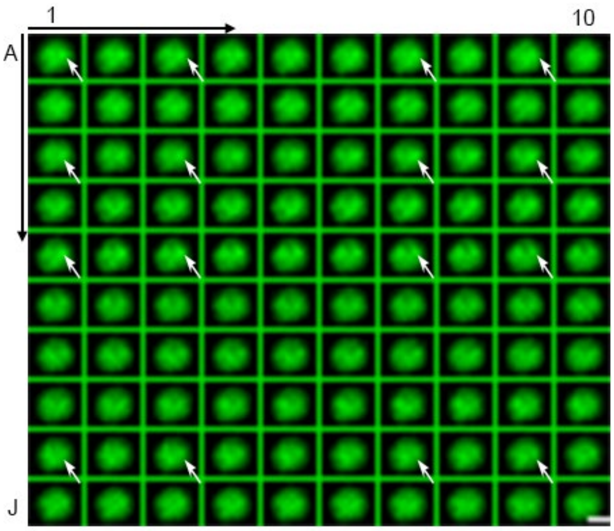

FIG S1. A montage of 100 frames (A1 to J10) of *B. cereus* spore 2 in Fig. 1. Each frame was acquired with 488 nm excitation light and at 535 nm emission light with an exposure time of 50ms and no delay interval. The scale bar is 1 µm.

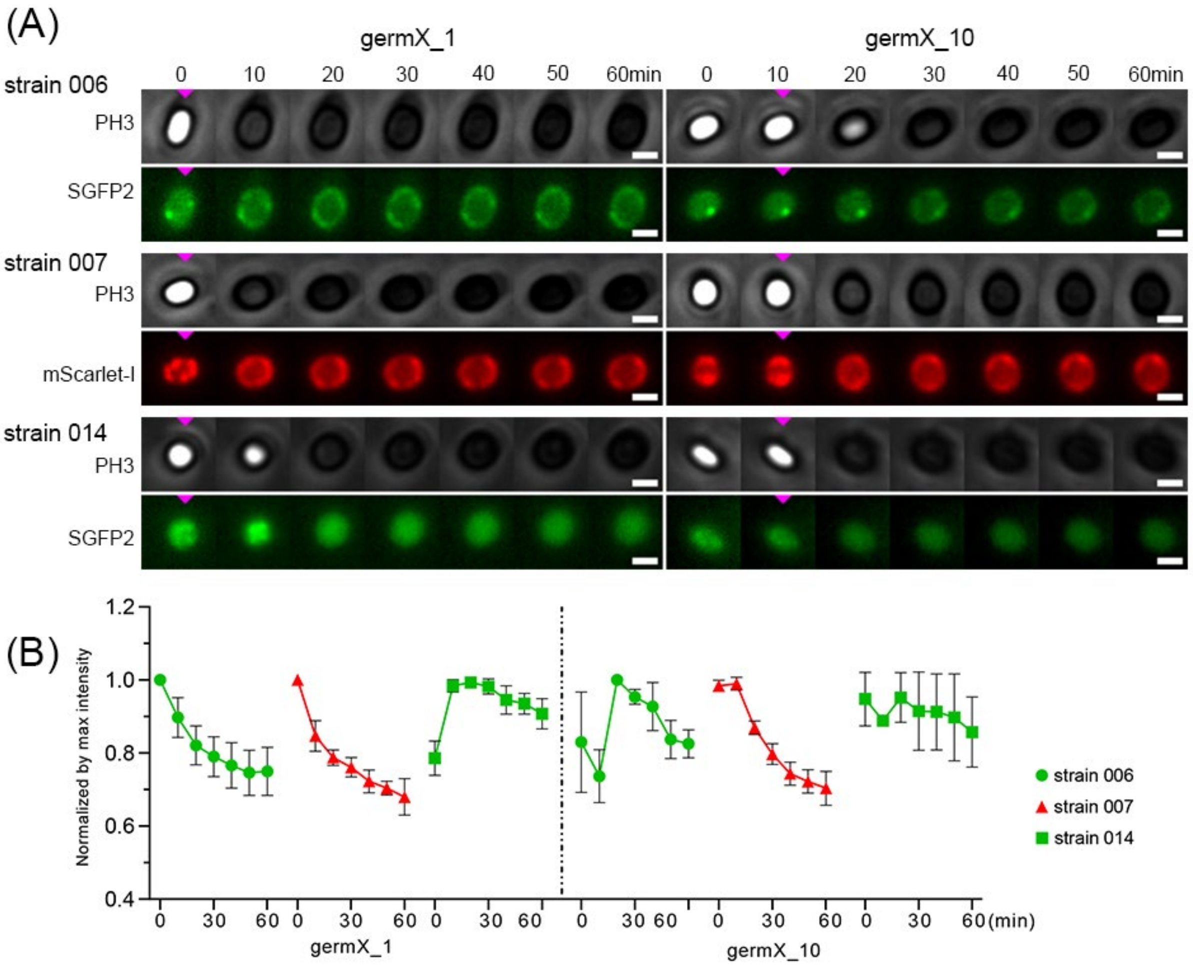

FIG S2. Dynamic changes in germinated spores of *B. cereus* strains 006, 007, and 014. Panel A, visualization of dynamics of two representative germinated spores of each strain at 10 min intervals over 60 min. Channels PH3 (phase contrast) and SGFP2 in the top layer are the groups germX_1 and germX_10 in spores of *B. cereus* strain 006. Channels PH3 and mScarlet-I in the middle layer are the groups germX_1 and germX_10 in spores of *B. cereus* strain 007. Channels PH3 and SGFP2 in the bottom layer are the groups germX_1 and germX_10 in spores of *B. cereus* strain 014. PH3, phase contrast. The pink triangles indicate the initiation of spore germination. The scale bar is 1 μm. Panel B, the line charts of SGFP2 (green) or mScarlet-I (red) channel intensities. Left column: group germX_1; right column: group germX_10. Data are shown as the mean with SD from three independent experiments. The numbers of analyzed germinated spores of *B. cereus* strains 006, 007 and 014 are given in Table S2. The statistical analyses of results are given in Table S4.
